# Acupuncture for irritable bowel syndrome: primary care based pragmatic randomised controlled trial

**DOI:** 10.1186/1471-230X-12-150

**Published:** 2012-10-24

**Authors:** Hugh MacPherson, Helen Tilbrook, J Martin Bland, Karen Bloor, Sally Brabyn, Helen Cox, Arthur Ricky Kang’ombe, Mei-See Man, Tracy Stuardi, David Torgerson, Ian Watt, Peter Whorwell

**Affiliations:** 1 Department of Health Sciences, University of York, York, UK; 2 Wythenshawe Hospital, Manchester, UK

**Keywords:** Acupuncture, Irritable bowel syndrome, Randomised controlled trial, Primary care

## Abstract

**Background:**

Acupuncture is used by patients as a treatment for irritable bowel syndrome (IBS) but the evidence on effectiveness is limited. The purpose of the study was to evaluate the effectiveness of acupuncture for irritable bowel syndrome in primary care when provided as an adjunct to usual care.

**Methods:**

Design: A two-arm pragmatic randomised controlled trial.

Setting: Primary care in the United Kingdom.

Patients: 233 patients had irritable bowel syndrome with average duration of 13 years and score of at least 100 on the IBS Symptom Severity Score (SSS).

Interventions: 116 patients were offered 10 weekly individualised acupuncture sessions plus usual care, 117 patients continued with usual care alone.

Measurements: Primary outcome was the IBS SSS at three months, with outcome data collected every three months to 12 months.

**Results:**

There was a statistically significant difference between groups at three months favouring acupuncture with a reduction in IBS Symptom Severity Score of −27.43 (95% CI: –48.66 to −6.21, p = 0.012). The number needed to treat for successful treatment (≥50 point reduction in the IBS SSS) was six (95% CI: 3 to 17), based on 49% success in the acupuncture group vs. 31% in the control group, a difference between groups of 18% (95% CI: 6% to 31%). This benefit largely persisted at 6, 9 and 12 months.

**Conclusions:**

Acupuncture for irritable bowel syndrome provided an additional benefit over usual care alone. The magnitude of the effect was sustained over the longer term. Acupuncture should be considered as a treatment option to be offered in primary care alongside other evidenced based treatments.

**Trial Registration:**

Current Controlled Trials ISRCTN08827905

## Background

General practitioners [1] and patients [2] have expressed their concerns regarding the adequacy and effectiveness of current treatments for irritable bowel syndrome (IBS). In a UK survey, 5% of patients receiving acupuncture reported having consulted primarily for gastrointestinal complaints, the most common being IBS [3]. However, the evidence on acupuncture for IBS is weak. A Cochrane review of six trials, with a median sample size of 54, found insufficient evidence to determine if acupuncture is an effective treatment for IBS [4]. No evidence of an acupuncture effect beyond placebo was found in two subsequent trials which controlled for non-specific effects [5,6].

Apart from three Chinese-based trials in the Cochrane review, none of the above trials were designed to evaluate the overall effect of acupuncture, *i.e.* an evaluation of the combined specific effect of acupuncture and non-specific effects associated with placebo. Moreover all of the trials delivered acupuncture in a way that the intervention was constrained, such that acupuncturists were not delivering acupuncture in a similar way to how they would do so normally in routine care. None of the trials were reported as based in primary care, instead they were reported as based in hospital settings [5-11] or settings were not reported [12,13]. Given this evidence gap, and the uncertainty outlined in the Cochrane review, further investigation of acupuncture for irritable bowel syndrome is merited [4].

In this study we designed an open pragmatic randomised controlled trial, in which we account for both the temporal effects associated with any spontaneous recovery and the effects of regression-to-the-mean, with the aim of capturing the overall impact of acupuncture when provided as a referral option in primary care. Our focus on a primary care setting complements previous studies conducted in secondary care [5-11]. We aimed to provide acupuncture as close as possible to how it would normally be provided in routine care, with the incorporation of explanations and lifestyle advice based on acupuncture theory in a way that is integral to practice [14,15]. This focus on acupuncture as a complex intervention precludes the possibility of a feasible and valid sham to control for all interacting components in a two-arm trial [16,17]. Our design mirrors that used in a trial of acupuncture for chronic low back pain which influenced policy in the UK; the study was central to the decision by the National Institute for Health and Clinical Excellence (NICE) to recommend 10 sessions of acupuncture for persistent low back pain [18].

Based on this rationale, our research therefore aims to provide practical comparative data on acupuncture for irritable bowel syndrome that is relevant to primary care and will be immediately applicable to patients and providers. This design provides the evidence needed to assist policy and decision-makers when considering acupuncture as a referral option for patients with irritable bowel syndrome.

## Methods

### Design overview

Building on our pilot study (ISRCTN32823720) [19], we conducted a parallel-arm randomised controlled trial to determine the effectiveness of acupuncture plus usual care compared to usual care alone for the treatment of IBS based on a published protocol [20]. We received ethics approval from the York NHS Research Ethics Committee (08/H1311/66) in 2008. A cost-effectiveness analysis is reported separately [21].

### Settings and participants

We recruited patients from the databases of five general practices. The GP practices identified potential participants aged 18 or older who had consulted their GP and been diagnosed with IBS, and coded accordingly on the practice database. A letter was sent to all potentially eligible participants, inviting them to complete a baseline questionnaire and consent form and return these to the York Trials Unit. We excluded patients who did not speak English, who had a current diagnosis of haemophilia, hepatitis, HIV, or were receiving cancer care, had had major gastrointestinal surgery in the previous six months, were pregnant, had a history of psychosis or substance abuse, or were receiving acupuncture at the time. Patients were recruited if they provided informed consent and scored 100 or more on the IBS Symptom Severity Score (SSS) [22].

### Randomisation

We randomised participants equally to receive either a short course of traditional acupuncture plus usual care or usual care alone. The randomisation sequence was computer generated at the University of York by an independent data manager at the York Trials Unit, and fully concealed from the research and administrative staff who then informed participants of their allocation by telephone and subsequently by letter.

### Interventions

Nine professional acupuncturists provided the acupuncture, and all were registered with the British Acupuncture Council with at least three years’ post qualification experience, and were working at independent clinics. Their practice style was based on principles of Traditional Chinese Medicine or “TCM”. Acupuncturists provided up to 10 sessions of acupuncture, adapted from a previously tested protocol [19], which allowed explanations and life-style advice based on acupuncture theory and clinical judgement [16]. While actual selection of points was individualised for patients and allowed to change over time, there was standardisation of the function of the acupuncture based on the theoretical frameworks used, an approach proposed for maintaining interventional integrity when delivering complex interventions [23].

All patients remained under the care of their general practitioner, and received usual care according to need. We documented usual care, for both NHS and non-NHS treatments, in both groups at three, six, nine and 12 months.

### Outcomes and follow-up

Our primary outcome measure was the IBS Symptom Severity Score (IBS SSS). This is scored from 0 to 500 (<75 = no IBS, 75–175 = “mild” case, 175–300 = “moderate” and 300+ = “severe”), and it has been validated for use in IBS patients [22]. Our primary end-point was at three months, with further follow-ups at six, nine and 12 months. We recorded participants’ baseline belief in acupuncture, expectations that acupuncture might help their IBS and treatment preferences.

Our secondary outcome measures were the IBS Non-Colonic Symptom Score (which includes lethargy & tiredness, “wind”, backache, and other symptoms [24], and SF-12 to evaluate patients’ health related quality of life [25]. EQ-5D was the primary outcome for the economic study [26]. Outcomes at follow-up points were sought by postal questionnaire, and where that failed to elicit a response, the main outcome measure was sought by telephone.

### Statistical analysis

In our pilot study [19], we calculated that for a 50 point difference on the IBS SSS, and an adjusted standard deviation to be 105 points, we needed to recruit a sample size of 94 patients per arm to have 90% power and a significance level of 0.05. To allow for loss to follow up of a similar proportion as observed in the pilot at three months (13%), our trial required at least 220 patients. In the full-scale trial we used analysis of covariance on an intention-to-treat basis to evaluate changes between groups for the primary outcome of the IBS SSS at three months, while adjusting for baseline scores and general practice. The statistical analysis was conducted using SAS version 9.2 and STATA version 10 using a 2-sided 5% significance level. We took a 50 point improvement [22] or more on the IBS SSS between baseline and three months as a “success” for our number-needed-to-treat analysis. We used similar analyses to explore between-group differences on the IBS SSS at other time points, and on secondary outcome measures. We used linear regression to explore whether expectation, belief and preference were treatment effect modifiers.

## Results

### Recruitment and follow-up of participants

**Figure 1:**
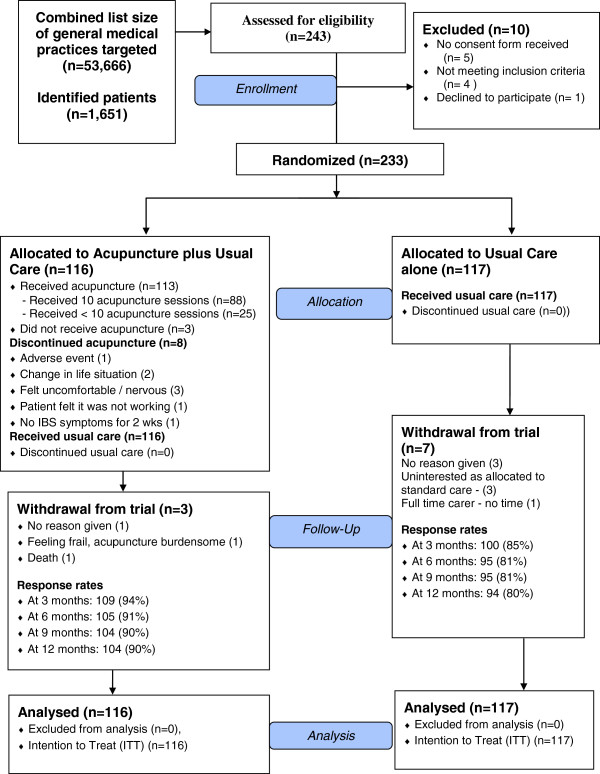
CONSORT flow diagram.

The trial recruited patients from five general practices (total list size of 53,666) between November 2008 and June 2009. GP practices identified and invited 1,651 potentially eligible patients to participate. Of the 243 patients who responded with baseline documentation to the York Trials Unit, 233 were recruited, see Flow Chart in Figure 1. The average number recruited per general practice was 47 (range 24 to 75).

### Baseline characteristics

Patients’ mean age at baseline was 44, and 81% were women, see Table 1 for full baseline details. The average duration of their IBS was 13 years. In terms of potential treatment effect modifiers, 56% believed acupuncture worked, 29% expected that acupuncture would help their IBS, 76% had a preference to be allocated to acupuncture, and 39% received their preferred treatment option.

**Table 1:** Baseline patient characteristics

**Characteristic**	**Acupuncture (n = 116)**	**Usual care (n = 117)**	**Overall (n = 233)**
**Age**			
Mean (SD)	44.28 (14.31)	42.68 (14.79)	43.47 (14.54)
Median (min to max)	43.81 (21.33 to 78.29)	42.00 (19.51 to 74.47)	42.69 (19.51 to 78.29)
**Gender**			
Male, n (%)	21 (18.10)	24 (20.51)	45 (19.31)
**Education (full time)**			
Yes (%)	5 (4.31)	4 (3.42)	9 (3.86)
**Work (full time)**	**(n = 90)**	**(n = 81)**	**(n = 171)**
Yes (%)	57 (49.14)	63 (53.85)	120 (51.50)
**Work (Not full time)**	**(n = 54)**	**(n = 51)**	**(n = 105)**
Work part-time	31 (26.72)	19 (16.24)	50 (21.16)
Currently looking for work	1 (0.86)	4 (3.42)	5 (2.15)
Permanently unable to work	4 (3.45)	0 (0.00)	4 (1.72)
Looking after home/family	4 (3.45)	10 (8.55)	14 (6.01)
Retired	11 (9.48)	16 (13.68)	27 (11.59)
Other	3 (2.59)	2 (1.71)	5 (2.15)
**Belief:** In general, do you think acupuncture can work?			
Yes (%)	61 (52.59)	69 (58.97)	130 (55.79)
No (%)	1 (0.86)	0 (0.00)	1 (0.43)
Don’t know (%)	54 (46.55)	48 (41.03)	102 (43.78)
**Expectation:** Do you think acupuncture may help your IBS?			
Yes (%)	31 (26.72)	37 (31.62)	68 (29.18)
No (%)	1 (0.86)	1 (0.85)	2 (0.86)
Don’t know (%)	84 (72.41)	79 (67.52)	163 (69.96)
**Preference 1:** Which treatment do you prefer?			
Acupuncture (%)	90 (77.59)	88 (75.21)	178 (76.39)
Standard care (%)	0 (0.00)	1 (0.85)	1 (0.43)
Don’t mind either (%)	26 (22.41)	28 (23.93)	54 (23.18)
**Preference 2:** Did you get your treatment preference?			
Got what I preferred (%)	90 (77.59)	1 (0.85)	91 (39.06)
Did not get what I preferred (%)	0 (0.00)	88 (75.21)	88 (37.77)
Did not have preference (%)	26 (22.41)	28 (23.93)	54 (23.18)
**Irritable Bowel Syndrome Symptom Severity Score (IBS SSS):**			
Mean (SD)	280.00 (81.34)	277.17 (71.50)	278.59 (76.47)

### Acupuncture provision within the acupuncture arm of the trial

There were seven female and two male acupuncture practitioners with post-qualification experience ranging from 3 to 25 years (mean 11). Practitioners provided acupuncture for 113 patients, averaging 12 patients each (range 3 to 22), covering 1016 treatments. The reasons for patient non-attendance and discontinuation are provided in Figure 1. Patients received an average of 9 sessions, with 88 patients (76%) receiving the full course, and the average course of treatment was completed within 12 weeks. Sessions were commonly 45 minutes duration and provided at weekly intervals. In Table 2 we provide a summary of the treatment details according to the STRICTA requirements [27] and a full report is published separately [28].

**Table 2:** **Details of the acupuncture treatment in items structured according to the STRICTA**[27]**reporting guidelines, an official extension to CONSORT**

**STRICTA item**	**Details of acupuncture treatment within the trial**
1. a) Style	Traditional Chinese Medicine (TCM).
b) Reasoning for treatment provided	Systematic review by Lim [4] and pragmatic design to evaluate acupuncture as provided in routine care.
c) Variation	Individualized treatments using common TCM theoretical frameworks: primarily *zang-fu syndromes* (used with 99% of patients), and also *qi, blood and body fluids*, *eight principles*, *five elements*, and *external pathogenic factors*.
2. a) Number of needles per treatment	On average 14 needles were inserted per session (range: 4 – 23) using an average of seven point locations (range 5 to 9).
b) Names	126 different points were used. A common core of points, LI 4, LR 3, ST 36, SP 6, were used in over 50% of treatments.
c) Depth of insertion	Average depth was 1.5 cm (range: 0.2 – 5 cm).
d) Response sought	The response sought varied, most commonly *de qi.*
e) Needle stimulation	Manual.
f) Retention	Average 20 min (range: few seconds to 30 min).
g) Needle type	Most common length was 25 mm (range: 15–40 mm) and diameter 0.20 mm (range: 0.30 to 0.16 mm).
3. a) Number of sessions	Patients were offered 10 sessions and completed an average of 9 sessions.
b) Frequency & duration	Usually once a week over 12 weeks.
4. a) Other components of treatment	Acupuncturists were allowed to use cupping, moxa, brief tui-na, brief acupressure, breathing, and ear seeds. The most commonly used was moxa (used with 13% of patients), brief *tui na* (9%) and brief acupressure (6%). Herbs & magnets were prohibited. Acupuncturists were allowed to provide lifestyle advice as part of the patient’s treatment consistent with their routine practice, with a restriction against probiotics. In total 68% of patients received lifestyle advice, most commonly diet (56%), stress reduction and relaxation (24%) and exercise (6%).
b) Setting and context	Provision or treatments in independent clinics. Acupuncturists encouraged to practice as closely as possible as they normally would.
5. Participating acupuncturists	British Acupuncture Council members, with more than three years post-qualification experience. Predominant treatment style: Traditional Chinese Medicine.
6. Control or comparator interventions	Patients in both groups continued to receive their usual care from their general practitioner, as well as over-the-counter treatments according to need. This allowed us to evaluate the impact of acupuncture as an adjunct to usual care. A summary of usual care actually received in both arms are provided in the main text.

### Usual care provision across both arms of the trial

We mapped the provision of usual care in both groups, which was provided or purchased according to need, and found differences in utilisation were minimal (see Additional file 1). For example in the first three months 59/83 (69%) of patients in the acupuncture group consulted at the their GP practice, compared to 72% of patients in the usual care group, with patient consulting on average 2.08 times and 2.25 times respectively, with 68% and 69% of these visits being related to IBS respectively. Over the first three months, 65% of the acupuncture group received prescription medication compared to 61% of the usual care group, with antispasmodics drugs, antidiarrhoeal drugs and laxatives making up a relatively small proportion of this utilisation (Additional file 1). Non-prescription medication was used by 58% in the acupuncture group compared to 60% in the usual care group.

### Patient outcomes

**Figure 2:**
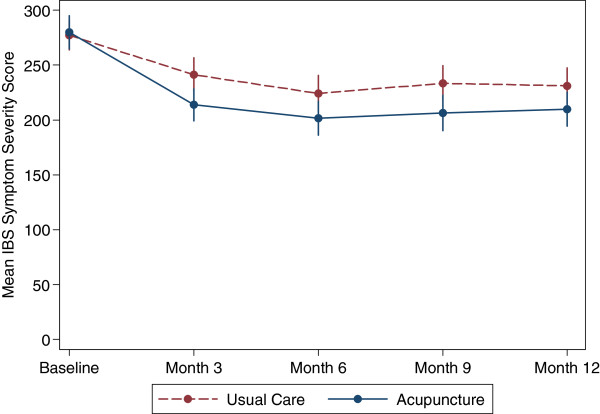
Longitudinal evolution of the mean IBS symptom severity scores against time of follow up by acupuncture and control groups.

Table 3 shows the results from the linear regression model providing IBS Symptom Severity Scores at baseline, 3, 6, 9 and 12 months, as illustrated in Figure 2. The between-group comparison showed a statistically significant reduction in symptoms in those allocated to acupuncture at three months: –27.43 (95% CI: –48.66 to −6.21, p = 0.012). The effect size could be described as small to moderate (Cohen’s *d =* 0.36). Treatment was assumed “successful” (≥50 points reduction on IBS SSS) in 57/116 (49%) of patients in the acupuncture group and 36/117 (31%) in those receiving usual care alone, a difference of 18% (95% CI: 6% to 31%). The number needed to treat was 6 (95% CI: 3 to 17), which means that for roughly every 6 participants treated with acupuncture, you will see one participant with an improvement equal to or greater than 50 in their baseline IBS SSS score.

**Table 3:** Results of linear regression models fitted at each separate time point on the IBS Symptom Severity Score

**Variable**	**Baseline Mean (SD)**	**Month 3 (95% CI)**	**Month 6 (95% CI)**	**Month 9 (95% CI)**	**Month 12 (95% CI)**
**Primary analysis: IBS Symptom Severity Score**					
Acupuncture group	280.00 (81.34)	213.78 (199.31 to 228.25)	201.60 (186.08 to 217.12)	206.40 (190.39 to 222.41)	209.79 (194.56 to 225.03)
Usual care group	277.17 (71.50)	241.21 (225.69 to 256.73)	224.19 (207.91 to 240.48)	233.40 (217.21 to 249.59)	231.12 (214.81 to 247.44)
Between group differences in means (acupuncture-usual care)	-	−27.43 (−48.66 to −6.21)	−22.59 (−45.11 to −0.08)	−27.00 (−49.77 to −4.23)	−21.33 (−43.66 to 1.00)

At each time point, predicted means and their 95% confidence intervals from the fitted model are presented except at baseline where the raw means and their standard deviation are presented.

The difference on the IBS SSS scores between groups subsequent to 3 months, adjusted for baseline, showed a statistically significant reduction at 6 months [−22.59] and 9 months [−27.0], followed by a near significant reduction at 12 months [−21.33], see Table 3 for confidence intervals. We found that patient expectation and belief were not treatment effect modifiers. Nor was preference, whether on the basis of comparing groups who preferred acupuncture vs. those who didn’t mind, or when comparing those who got their preference vs. those who did not. We found a statistically significant reduction favouring the acupuncture group for the IBS Non-Colonic Score only at three months, [−15.24 (95% CI: –29.91 to −0.57)], but no significant differences in the physical and mental component scores on the SF-12 at any time point (see Additional file 2).

### Adverse events

No serious adverse events associated with acupuncture were reported by acupuncturists in the logbooks or by patients in follow-up questionnaires. Non-serious adverse events associated with acupuncture were reported by eleven patients: eight experienced pain from the needles (three of the eight also reported nerve pain, bruising or fatigue), one had bruising from the needles, one had a slight burn from a moxibustion stick, and one had itchy and swollen skin where one of the needles had been inserted.

## Discussion

### Summary of main findings

For patients with chronic irritable bowel syndrome, our study showed that acupuncture provided as an adjunct in primary care is associated with a small yet statistically significant clinical reduction in symptoms at three months. This was reflected in 49% of patients in the acupuncture group and 31% in the usual care group improving by more than 50 points in their IBS SSS score, with six being the number-needed-to-treat. This effect was largely sustained through to 12 months. We also found acupuncture for this group of patients had a high level of acceptability, as shown by the willingness by patients to attend for acupuncture once they had commenced treatment, completing on average 9 out of the 10 weekly acupuncture sessions that were available.

### Strengths and limitations

This study is the first pragmatic randomised controlled trial of acupuncture for IBS in primary care. The design ensured that we controlled for temporal effects associated with spontaneous recovery and regression-to-mean effects associated with having a minimum cut-off on our IBS SSS scale. The trial had an adequate sample size, and was conducted rigorously at all stages, with unambiguously concealed allocation to prevent subversion of the randomisation. Of the 1,651 potentially eligible patients identified by GP practices that were invited to participate, 243 patients responded, a rate that is typical of trials in primary care that use a database method for recruitment. We used validated outcomes measures, with response rates at 3, 6, 9 and 12 months that were reasonably high, at over 80 per cent in the control group and over 90 per cent in the intervention group. We have documented that there was reasonable equivalence in usual care received in both groups at three months, which leads us to cautiously ascribe any differences between groups to the treatment provided by acupuncturists. We explored the impact of variables that are potentially associated with placebo effects, namely patients’ beliefs, expectations and treatment preferences [29], but they were not found to be treatment effect modifiers.

There is likely to be a contribution to the overall benefit we observed due to the additional attention received in the acupuncture group resulting from the contact time with practitioners in the acupuncture arm. We did not control for this bias, which would have required a trial arm with a practitioner-led intervention in order to control for time and attention. The patients in our study were not blind to their group allocation, as would have been the case in a sham-controlled trial. A comparator trial arm with a sham control would have had the limitation that the verum acupuncture would need to be constrained to minimise variability in patient experience between the verum and sham acupuncture arms. There is a trade-off between seeking to know more about the contribution each of the components might (or might not) contribute to any putative benefit and seeking to know more about the impact of acupuncture as a package of care that more closely reflects routine practice. A third trial arm was outside of the scope of our funding support.

The study population was made up of those who have consulted with predominantly “moderate” IBS symptoms in primary care (average IBS SSS score of 279), have experienced these symptoms for an average of 13 years and have not experienced sufficient symptom reduction from their usual care so that they are continuing to seek care. Therefore generalising the results to patients who are consulting their GP for the first time, and who therefore have not experienced usual GP care and its impact on symptoms, must be done with caution. The trial was not powered to determine whether any of the sub-groupings of patients with IBS symptoms (for example based on severity) would have fared better or worse.

### Comparison with existing studies

Our study had the longest duration in terms of collecting outcome data through to 12 months after randomisation. By contrast, most other studies have been conducted within hospital based settings, with more narrowly defined populations, smaller sample sizes and shorter term follow-ups [4]. The level of acceptability of acupuncture treatments among patients was high (only three patients out of 116 did not start treatment and 88 (76%) completed their full course of treatment, with an overall average of nine sessions attended out of 10) compared to a trial of cognitive behavioural therapy for IBS in which fewer than half of patients were considered by the therapist to have completed therapy, with 41% either declining therapy or dropping out [30].

Components of acupuncture are drawn from three categories: needling related components; non-needling components that are acupuncture-specific, such as explanations and lifestyle advice; and non-specific components that are generic to the therapeutic encounter, such as the patient-practitioner relationship [31]. Our trial is unique in that it was designed to include the second of these components, as well as the other two. However it was not designed to determine the extent to which each of these components of acupuncture contributed to the overall outcome. To establish a proportional contribution of a component of treatment would require a trial arm with a sham control, for example as has been done recently to explore the impact of the patient-practitioner relationship [10]. In their trial, sham acupuncture needling was used to control for the needling in verum acupuncture arm, which then allowed the impact of a variation in the intensity of patient-practitioner relationship to be measured. By contrast, our focus on acupuncture as a complex intervention with many interacting components meant that a feasible and valid sham control was problematic [16,17].

With regard to placebo effects among patients with IBS, there is some limited evidence from other studies that these effects appear to tail off after three months, according to a study that reviewed 27 randomised controlled trials of treatments for IBS, with individual trials showing no placebo response at 12 months [32]. The results from our trial, in contrast, provide some evidence of a sustained effect at 12 months.

### Implications for future research and clinical practice

Our trial focussed on those patients with long term symptoms at recruitment (average 13 years duration) for whom usual GP care was of limited benefit; however, exploring the potential impact of referral to acupuncture soon after an initial diagnosis of IBS would be useful. An appropriately powered study could determine whether any of the sub-groupings of patients with IBS symptoms (for example based on mild, moderate or severe categories) fared better or worse, and also characterize responders and non-responders to acupuncture. A clearer specification of optimal acupuncture treatment for this group of patients would be helpful. In particular it would be useful to determine if more than 10 acupuncture sessions would improve outcomes, as from the graph in Figure 2 the steep improvement of the acupuncture group appears to level off quite abruptly when treatment ceases. To separate out the impact of different components of treatment, a sham controlled trial design similar to that used by Kaptchuk and colleagues [10] could be used to determine whether or not the provision of explanations and life-style advice might (or might not) add to any overall effect, assuming that effects of different treatment components are additive rather than synergistic.

There is a need for a number of effective options for treatment in primary care, given the frustration experienced by some patients with existing treatments [2]. Our results showed that, on average, acupuncture is associated with a small to moderate reduction in symptoms in a population that has had IBS symptoms for many years. If a patient responds at three months, then this benefit is likely on average to be sustained at 12 months. The IBS Symptom Severity Score is now widely used in IBS research and has been translated into many languages. When used in assessing response to treatment, a reduction of 50 points is regarded as clinically significant and, therefore, a reduction of 70 points after 12 months in a group of primary care patients in whom symptoms have been refractory, suggests that this is a treatment option worth considering in such individuals, especially as the beneficial effects seem to be sustained. These data on acupuncture are highly relevant to the primary care context in the UK and have policy implications.

## Conclusion

In a rigorously conducted pragmatic randomised controlled trial, we have evaluated the effectiveness of acupuncture as a treatment for irritable bowel syndrome when offered as an adjunct to usual treatment in primary care. Acupuncture was found to significantly improve outcomes at three months, with the number needed to treat being six. We found some evidence of a sustained benefit over the longer term. Acupuncture should be considered as a potential treatment option in primary care alongside other evidence-based treatments.

## Abbreviations

IBS: Irritable Bowel Syndrome; NHS: National Health Service; NICE: National Institute for Health & Clinical Excellence; SSS: Symptom Severity Score.

## Competing interests

HM has a part-time clinical practice that includes acupuncture.

## Authors’ contributions

HM conceived the study and was the lead applicant and principal investigator for the trial. HT was the lead trial co-coordinator, JMB was a co-applicant for funding and supervising statistician for the trial, KB was a co-applicant for funding and advised on matters relating to health economics and policy, SB was the trial administrator, HC was one of trial coordinators, ARK was the trial statistician and conducted all the clinical analyses, MM contributed as a trial co-ordinator, TS designed aspects of the trial, steered the ethics approval process and collected data on treatments provided, DT was a co-applicant for funding and advised on trial methodology, IW was a co-applicant and advised on matters relating to primary care and adverse events, PW is a consultant gastroenterologist who was a co-applicant and advised on clinical aspects of the trial and made available the primary outcome measure for the trial. All authors have had full access to all of the data (including statistical reports and tables) in the study and can take responsibility for the integrity of the data and the accuracy of the data analysis, and have read and approved the final manuscript.

## Authors’ information

Helen Tilbrook, Research Fellow, York Trials Unit, Department of Health Sciences, University of York, York, YO10 5DD, UK

Martin Bland, Professor of Health Statistics, Department of Health Sciences, University of York, York, YO10 5DD, UK

Karen Bloor, Senior Research Fellow, Department of Health Sciences, University of York, York, YO10 5DD, UK

Sally Brabyn, Trial Support Officer, Department of Health Sciences, University of York, York, YO10 5DD, UK

Helen Cox, Research Fellow, York Trials Unit, Department of Health Sciences, University of York, York, YO10 5DD, UK

Arthur Ricky Kang'ombe, Biostatistician, York Trials Unit, Department of Health Sciences, University of York, York, YO10 5DD, UK

Mei-See Man, Research Fellow, Department of Health Sciences, University of York, York, YO10 5DD, UK

Tracy Stuardi, PhD Student, Department of Health Sciences, University of York, York, YO10 5DD, UK

David Torgerson, Director of York Trials Unit, Department of Health Sciences, University of York, York, YO10 5DD, UK

Ian Watt, Professor of Primary Care, Hull York Medical School, York, YO10 5DD, UK

Peter Whorwell, Professor of Medicine and Gastroenterology, Wythenshawe Hospital, Manchester M23 9LT, UK.

## Pre-publication history

The pre-publication history for this paper can be accessed here:

http://www.biomedcentral.com/1471-230X/12/150/prepub

## Supplementary Material

Additional file 1 Utilisation of usual care in both groups, with data on previous three months recorded by patients retrospectively at three-monthly intervals. Click here for file

Additional file 2 Secondary outcomes based on linear regression modelling at 3, 6, 9 and 12 months. Click here for file
