# Supplementary material for: Acupuncture for irritable bowel syndrome: primary care based pragmatic randomised controlled trial
Source: BMC Gastroenterol. 2012 Oct 24;12:150. doi: 10.1186/1471-230X-12-150 (PMC3556159; doi:10.1186/1471-230X-12-150)
Supplement: Additional file 1 — Utilisation of usual care in both groups, with data on previous three months recorded by patients retrospectively at three-monthly intervals. [file 1471-230X-12-150-S1.pdf]

Additional File 1: Utilisation of usual care in both groups, with data on previous three months recorded by patients retrospectively at three monthly intervals

	3 months		6 months		9 months		12 months	
	Usual Care	Acupuncture	Usual Care	Acupuncture	Usual Care	Acupuncture	Usual Care	Acupuncture
**Number of patients consulting at GP practice**	59/83	73/106	50/81	65/101	45/86	67/96	56/87	59/101
**Number of times consulting with:**								
**GP**								
N	53	71	42	62	44	60	55	51
Mean (sd)	2.25 (1.92)	2.08 (2.50)	2.31 (2.10)	1.82 (1.29)	2.27 (1.66)	2.08 (2.16)	2.11 (1.66)	2.20 (1.44)
Median (p25, p75)	2 (1, 2)	1 (1, 2)	1 (1, 3)	1 (1, 2)	2 (1, 3)	1.5 (1, 2)	2 (1, 3)	2 (1, 3)
**Practice nurse**								
N	30	36	29	42	30	39	28	31
Mean (sd)	0.63 (0.72)	1.00 (1.26)	0.72 (0.65)	0.74 (0.80)	0.93 (1.34)	0.82 (0.72)	0.96 (1.55)	1.74 (5.32)
Median (p25, p75)	0.5 (0, 1)	1 (0,1)	1 (0, 1)	1 (0, 1)	1 (0, 1)	1 (0, 1)	0.5 (0, 1)	1 (0, 1)
**Other**								
N	22	22	17	22	15	15	19	20
Mean (sd)	0.68 (1.46)	0.82 (2.17)	1.47 (3.30)	0.50 (1.34)	0.07 (0.26)	0.53 (0.83)	0.32 (0.95)	0.60 (0.75)
Median (p25, p75)	0 (0, 1)	0 (0, 1)	0 (0, 1)	0 (0, 0)	0 (0, 0)	0 (0, 1)	0 (0, 0)	0 (0, 1)
**Number of above visits related to IBS**								
N	57	72	48	62	42	63	51	55
Mean (sd)	0.54 (0.83)	0.51 (1.20)	0.38 (0.76)	0.48 (0.88)	0.81 (1.27)	0.41 (0.89)	0.45 (0.81)	0.71 (1.46)
Median (p25, p75)	0 (0, 1)	0 (0, 1)	0 (0, 0.5)	0 (0, 1)	0 (0, 1)	0 (0, 0)	0 (0, 1)	0 (0, 1)
**Number of patients admitted to hospital as an emergency**	4/83	2/106	2/81	1/99	2/86	6/96	3/84	5/101
**Number of times admitted as emergency**								
None	0/4	0/2	0/2	0/1	0/2	0/6	0/3	1/5
Once	3/4	1/2	2/2	1/1	2/2	4/6	3/3	3/5
Twice	1/4	1/2	0/2	0/1	0/2	1/6	0/3	1/5

	3 months		6 months		9 months		12 months	
	**Usual Care**	**Acupuncture**	**Usual Care**	**Acupuncture**	**Usual Care**	**Acupuncture**	**Usual Care**	**Acupuncture**
Missing	0/4	0/2	0/2	0/1	0/2	1/6	0/3	0/5
**Number of above admissions related to IBS**	0/4	0/2	0/2	0/1	0/2	1/5	0/3	0/5
**Number of patients admitted to hospital not as an emergency**	6/79	3/105	6/80	4/96	4/83	5/94	5/85	5/99
**Number of times admitted not as emergency**								
None	0/6	0/3	0/6	0/4	0/4	1/5	0/5	0/5
Once	6/6	0/3	6/6	4/4	2/4	4/5	5/5	4/5
Twice	0/6	2/3	0/6	0/4	2/4	0/5	0/5	1/5
Missing	0/6	1/3	0/6	0/4	0/4	0/5	0/5	0/5
**Number of above admissions related to IBS**								
None	6/6	1/3	6/6	2/4	3/4	4/5	4/5	4/5
Once	0/6	0/3	0/6	2/4	0/4	1/5	0/5	0/5
Twice	0/6	1/3	0/6	0/4	0/4	0/5	0/5	1/5
Missing	0/6	1/3	0/6	0/4	1/4	0/5	1/5	0/5
**Number of times seen by doctor at an outpatient clinic of a hospital**								
N	76	94	68	86	67	82	75	79
Mean (sd)	0.28 (0.67)	0.21 (0.51)	0.34 (0.92)	0.24 (0.61)	0.33 (0.82)	0.37 (0.90)	0.45 (0.84)	0.42 (0.76)
Median (p25, p75)	0 (0, 0)	0 (0, 0)	0 (0, 0)	0 (0, 0)	0 (0, 0)	0 (0, 0)	0 (0, 0)	0 (0, 1)
**Number of above visits related to IBS**								
None	14/15	7/15	11/13	7/12	9/14	12/15	16/20	18/21
Once	1/15	6/15	2/13	5/12	5/14	3/15	3/20	2/21
Twice	0/15	2/15	0/13	0/12	0/14	0/15	1/20	1/21
**Number of times seen any other**								

	3 months		6 months		9 months		12 months	
	Usual Care	Acupuncture	Usual Care	Acupuncture	Usual Care	Acupuncture	Usual Care	Acupuncture
**health professional from NHS:**								
**Acupuncturist**								
N	63	80	60	75	56	69	61	76
Mean (sd)	0.00 (0.00)	0.21 (1.36)	0.00 (0.00)	0.23 (1.46)	0.00 (0.00)	0.10 (0.49)	0.00 (0.00)	0.12 (0.77)
Median (p25, p75)	0 (0, 0)	0 (0, 0)	0 (0, 0)	0 (0, 0)	0 (0, 0)	0 (0, 0)	0 (0, 0)	0 (0, 0)
**Chiropractor or osteopath**								
N	64	83	61	73	57	67	61	73
Mean (sd)	0.58 (3.37)	0.16 (1.04)	0.11 (0.78)	0.10 (0.71)	0.02 (0.13)	0.00 (0.00)	0.00 (0.00)	0.00 (0.00)
Median (p25, p75)	0 (0, 0)	0 (0, 0)	0 (0, 0)	0 (0, 0)	0 (0, 0)	0 (0, 0)	0 (0, 0)	0 (0, 0)
**Other**								
N	67	80	63	72	59	71	65	77
Mean (sd)	0.40 (1.22)	0.58 (1.82)	0.46 (1.52)	0.32 (1.12)	0.46 (1.47)	0.14 (0.90)	0.46 (1.79)	0.34 (1.05)
Median (p25, p75)	0 (0, 0)	0 (0, 0)	0 (0, 0)	0 (0, 0)	0 (0, 0)	0 (0, 0)	0 (0, 0)	0 (0, 0)
**Number of above visits related to IBS**								
N	38	47	34	45	31	36	32	41
Mean (sd)	0.00 (0.00)	0.45 (1.77)	0.06 (0.34)	0.36 (1.80)	0.14 (0.59)	0.14 (0.59)	0.00 (0.00)	0.10 (0.37)
Median (p25, p75)	0 (0, 0)	0 (0, 0)	0 (0, 0)	0 (0, 0)	0 (0, 0)	0 (0, 0)	0 (0, 0)	0 (0, 0)
**Number of times consulted a private healthcare professional:**								
**Doctor**								
N	61	83	64	70	60	69	65	71
Mean (sd)	0.00 (0.00)	0.04 (0.24)	0.02 (0.13)	0.04 (0.20)	0.00 (0.00)	0.03 (0.17)	0.12 (0.76)	0.04 (0.26)
Median (p25, p75)	0 (0, 0)	0 (0, 0)	0 (0, 0)	0 (0, 0)	0 (0, 0)	0 (0, 0)	0 (0, 0)	0 (0, 0)
**Acupuncturist**								
N	61	81	65	76	59	75	64	74
Mean (sd)	0.10 (0.77)	0.19 (1.37)	0.14 (0.79)	0.66 (2.04)	0.17 (1.07)	0.41 (1.15)	0.09 (0.75)	0.34 (1.11)
Median (p25, p75)	0 (0, 0)	0 (0, 0)	0 (0, 0)	0 (0, 0)	0 (0, 0)	0 (0, 0)	0 (0, 0)	0 (0, 0)
**Chiropractor or osteopath**								

	3 months		6 months		9 months		12 months	
	**Usual Care**	**Acupuncture**	**Usual Care**	**Acupuncture**	**Usual Care**	**Acupuncture**	**Usual Care**	**Acupuncture**
N	64	83	66	70	63	68	67	71
Mean (sd)	0.31 (1.15)	0.16 (0.61)	0.26 (1.30)	0.16 (0.83)	0.14 (0.59)	0.09 (0.51)	0.22 (0.78)	0.06 (0.37)
Median (p25, p75)	0 (0, 0)	0 (0, 0)	0 (0, 0)	0 (0, 0)	0 (0, 0)	0 (0, 0)	0 (0, 0)	0 (0, 0)
**Other**								
N	61	79	62	68	53	70	59	72
Mean (sd)	0.20 (0.63)	0.19 (0.64)	0.23 (0.91)	0.19 (0.92)	0.13 (0.48)	0.39 (1.88)	0.17 (0.65)	0.53 (2.99)
Median (p25, p75)	0 (0, 0)	0 (0, 0)	0 (0, 0)	0 (0, 0)	0 (0, 0)	0 (0, 0)	0 (0, 0)	0 (0, 0)
**Number of above consultations related to IBS**								
N	40	54	42	58	36	48	40	55
Mean (sd)	0.15 (0.53)	0.48 (1.85)	0.38 (1.41)	0.93 (2.63)	0.39 (1.78)	1.02 (3.26)	0.20 (0.97)	1.00 (3.56)
Median (p25, p75)	0 (0, 0)	0 (0, 0)	0 (0, 0)	0 (0, 0)	0 (0, 0)	0 (0, 0)	0 (0, 0)	0 (0, 0)
**Number of patients using any prescription medication**	50/82	68/105	52/82	61/97	46/83	67/95	56/85	69/101
**IBS-related prescription medication**								
Antispasmodic drugs	11/82	9/105	11/82	7/97	14/83	6/95	20/85	9/101
Antidiarrhoeal drugs	5/82	9/105	3/82	5/97	3/83	5/95	2/85	4/101
Laxatives	4/82	13/105	2/82	12/97	4/83	12/95	6/85	12/101
**Number of patients using any non-prescription medication or supplement**	47/78	58/100	51/81	63/94	46/80	52/92	51/85	55/100
