# Supplementary material for: Acupuncture for irritable bowel syndrome: primary care based pragmatic randomised controlled trial
Source: BMC Gastroenterol. 2012 Oct 24;12:150. doi: 10.1186/1471-230X-12-150 (PMC3556159; doi:10.1186/1471-230X-12-150)
Supplement: Additional file 2 — Secondary outcomes based on linear regression modelling at 3, 6, 9 and 12 months. [file 1471-230X-12-150-S2.pdf]

Additional File 2: Secondary outcomes based on linear regression models at 3, 6, 9 and 12 months

Variable	Baseline Mean (std)	Month 3 (95% CI)	Month 6 (95% CI)	Month 9 (95% CI)	Month 12 (95% CI)
**Primary analysis: Symptom Severity Score**					
Acupuncture group	280.00 (81.34)	213.78 (199.31 to 228.25)	201.60 (186.08 to 217.12)	206.40 (190.39 to 222.41)	209.79 (194.56 to 225.03)
Usual care group	277.17 (71.50)	241.21 (225.69 to 256.73)	224.19 (207.91 to 240.48)	233.40 (217.21 to 249.59)	231.12 (214.81 to 247.44)
Between group differences in means (acupuncture-usual care)	-	-27.43 (-48.66 to -6.21)	-22.59 (-45.11 to -0.08)	-27.00 (-49.77 to -4.23)	-21.33 (-43.66 to 1.00)
**Secondary analyses:**					
**Non-colonic Symptom Score**					
Acupuncture group	208.15 (79.11)	176.37 (166.31 to 186.43)	176.96 (165.89 to 188.02)	182.47 (169.63 to 195.31)	183.20 (171.39 to 195.01)
Usual care group	208.43 (73.05)	191.61 (180.94 to 202.28)	185.88 (174.23 to 197.52)	186.24 (173.05 to 199.43)	188.51 (175.87 to 201.14)
Between group differences in means (acupuncture-usual care)	-	-15.24 (-29.91 to -0.57)	-8.92 (-24.99 to 7.15)	-3.77 (-22.18 to 14.65)	-5.31 (-22.61 to 11.99)
**SF-12 version 2**					
**Mental**					

**Component Score**					
Acupuncture group	41.66 (11.55)	44.50 (42.82 to 46.17)	44.55 (42.82 to 46.29)	45.34 (43.57 to 47.12)	44.81 (43.10 to 46.52)
Usual care group	42.66 (9.45)	43.36 (41.47 to 45.25)	43.61 (41.70 to 45.53)	43.48 (41.59 to 45.38)	44.11 (42.26 to 45.96)
Between group differences in means (acupuncture-usual care)		1.14 (-1.38 to 3.66)	0.94 (-1.65 to 3.53)	1.86 (-0.75 to 4.46)	0.70 (-1.82 to 3.22)
**Physical Component Score**					
Acupuncture group	48.33 (9.13)	49.20 (48.11 to 50.30)	49.71 (48.48 to 50.93)	47.95 (46.64 to 49.25)	48.39 (47.04 to 49.74)
Usual care group	49.40 (8.22)	49.19 (47.96 to 50.42)	48.33 (46.99 to 49.67)	49.30 (47.91 to 50.68)	47.93 (46.48 to 49.39)
Between group differences in means (acupuncture-usual care)		0.0092 (-1.64 to 1.66)	1.37 (-0.44 to 3.19)	-1.34 (-3.25 to 0.56)	0.46 (-1.52 to 2.44)

At each time point, predicted means and their 95% confidence intervals from the fitted model are presented except at baseline where the raw means and their standard deviation are presented.
